# The Late Dr. H, G. Sutton

**Published:** 1891-06-20

**Authors:** 


### The Late Dr. H. G. Sutton.

A CORRESPONDENT writes: On Friday, June 12th, at St. Nicholas Church, Sevenoaks, Dr. Henry Gawen Sutton, late Physician of the London Hospital, was laid to rest. His work had identified him with the suffering poor of the East-end, to whom he has so nobly devoted the greater part of his life. None worked harder, and no one has done more permanent benefit to the poor and others identified with East London. Dr. Sutton was originally a student of Guy's Hospital, and his work in checking the cholera epidemic of 1866 was recognised and rewarded by his appointment as Assistant Physician of the London Hospital. Few medical men who have passed through the School attached to the great East-end charity do not recognise the deep obligation they all owe to the clinical teaching of their late Professor and teacher of pathology. To the House Committee his advice in regard to the management of the Institution was often of the greatest value. Among the wreaths were the following: "In affectionate remembrance," "From the members of the House Committee of the London Hospital, who fully appreciate the great loss sustained by the institution to which Dr. Sutton has so nobly devoted the greater part of his life." A large wreath of wild flowers bore the inscription, "From men and women who have known Dr. Sutton at the London Hospital, and to whom he has been and always will be a help and a guide." The House Physicians of Dr. Sutton sent a laurel wreath, "He needs no other rosary whose thread of life is strung with beads of thought and love." Amongst those present to pay respect to the deceased Physician were Dr. Wilks, of Guy's Hospital; Dr. Starkey, of St. Thomas's; Dr. Savage, Dr. Hughtings-Jackson, Mr. Waren Tay, Dr. Stephen Mackenzie, Dr. Turner, Dr. Herman, Dr. Lewers, Mr. Openshaw, Mr. A. Druce, of the House Committee; Mr. W. J. Nixon, Mr. G. Q. Roberts, and many House Physicians.

